# Cost-effectiveness of ambroxol in the treatment of Gaucher disease type 2

**DOI:** 10.1515/med-2024-0970

**Published:** 2024-05-21

**Authors:** Miloš N. Milosavljević, Medo Gutić, Vladimir Janjić, Slađana Veselinović, Milan Djordjić, Radenko Ivanović, Jovana Milosavljević, Slobodan M. Janković

**Affiliations:** Department of Pharmacology and Toxicology, Faculty of Medical Sciences, University of Kragujevac, 34000, Kragujevac, Serbia; Department of Psychiatry, Faculty of Medical Sciences, University of Kragujevac, 34000, Kragujevac, Serbia; Department of Communication Skills, Ethics and Psychology, Faculty of Medical Sciences, University of Kragujevac, 3400, Kragujevac, Serbia; University Hospital Foča, 73300, Foča, Republic of Srpska, Bosnia and Herzegovina; Faculty of Medicine in Foča, University of East Sarajevo, 73300, Foča, Republic of Srpska, Bosnia and Herzegovina; Department of Anatomy, Faculty of Medical Sciences, University of Kragujevac, 34000, Kragujevac, Serbia

**Keywords:** Gaucher diseases, ambroxol, cost-effectiveness, insurance, health

## Abstract

**Objective:**

Our aim was to compare the costs and efficacy of ambroxol in combination with imiglucerase with the costs and efficacy of imiglucerase only in the treatment of Gaucher disease type 2 (GD2) in the socio-economic settings of the Republic of Serbia, an upper-middle-income European economy.

**Methods:**

The perspective of the Serbian Republic Health Insurance Fund was chosen for this study, and the time horizon was 6 years. The main outcomes of the study were quality-adjusted life years gained with ambroxol + imiglucerase and comparator, and direct costs of treatment. The study was conducted through the generation and simulation of the Markov chain model. The model results were obtained after Monte Carlo microsimulation of a sample with 1,000 virtual patients.

**Results:**

Treatment with ambroxol in combination with imiglucerase was cost-effective when compared with imiglucerase only and was associated with positive values of net monetary benefit regardless of the onset of the disease. Such beneficial result for ambroxol and imiglucerase combination is primarily driven by the low cost of ambroxol and its considerable clinical effectiveness in slowing the progression of neural complications of GD2.

**Conclusion:**

If ambroxol and imiglucerase are used in combination for the treatment of GD2, it is more cost-effective than using imiglucerase alone.

## 1 Introduction

Gaucher disease is caused by the accumulation of glucocerebroside in lysosomes due to the lack of the glucocerebrosidase enzyme; it is inherited autosomal recessively. The disease has three phenotypic forms, designated as type 1, type 2, and type 3 [1]. In type 1, which is much more common (about 94% of all patients with Gaucher disease have this type of disease), accumulation of glucocerebroside predominantly occurs outside the central nervous system, in organs rich in reticuloendothelial cells – liver, spleen, bone marrow, and lungs. Type 1 is also called the non-neuronal form of the disease, because the nervous system is not affected by pathological changes. In types 2 and 3, which are called by one name the neuronal form of Gaucher disease, the primary damage is to the central nervous system, while the parenchymatous organs are affected little or not at all [2].

When it comes to types 2 and 3, i.e., the neuronal form of the disease, they differ from each other in terms of the type of genetic disorder and the severity of the clinical picture. Type 2 is also called the acute form, because neurological disorders develop quickly, so patients rarely live longer than 5 years. Type 3 is also called the chronic form, and with it, the disease progresses much more slowly, so patients live longer [3]. With type 2, we additionally distinguish two subgroups, formed on the basis of the onset of the disease. The first group consists of patients in whom the disease begins with signs and symptoms already visible at birth, and in the second group, the disease begins in infancy, that is, during the first year of life. While for types 1 and 3, there is a causal therapy that has shown effectiveness (enzyme replacement therapy, imiglucerase, velaglucerase, or taliglucerase), for type 2, it is mostly ineffective [4,5]. A few years ago, in one pilot study [6], and then in several case reports, a positive effect of ambroxol on neurological disorders in type 2 was shown, when it was used together with enzyme replacement therapy (which prevents the progression of pathological changes in parenchymatous organs). Ambroxol is the so-called pharmacological chaperone, which protects damaged glucocerebrosidase molecules from degradation and thus prolongs their effect. Additional clinical studies are needed to determine the true effect of ambroxol, and it is necessary to look at the cost-effectiveness ratio of the drug.

Our aim was to compare the costs and efficacy of ambroxol in combination with imiglucerase with the costs and efficacy of imiglucerase only in the treatment of Gaucher disease type 2 (GD2) in the socio-economic settings of the Republic of Serbia, an upper-middle-income European economy.

## 2 Methods

The health economic analysis plan that the authors developed before the study’s start was followed throughout the investigation. A full description of the study plan is available at: https://drive.google.com/drive/folders/1emX2mamfMqmrKd6c0AKdvRJiq8JKuwGA?usp=share_link.

The study setting was the healthcare system of the Republic of Serbia, which is an upper-middle-income European economy according to the World Bank [7]. Patients with GD2 of both sexes, with the disease beginning at birth or during infancy, represented our study population. We examined the cost-effectiveness of two therapeutic options for the treatment of GD2: (1) experimental, off-label combination of ambroxol and imiglucerase and (2) monotherapy of imiglucerase, the only approved causal therapy of GD2 until now [8]. The analysis was conducted from the perspective of the Republic Fund for Health Insurance (RHIF) of the Republic of Serbia [9]. We have defined the horizon as a period of 6 years, with cycles of one month each, according to the natural course of GD2 and the fact that almost none of the patients survive more than 5 years [10]. Table 1 shows the model inputs.

**Table 1:** The model inputs

Input parameter	Base case value with variability (±SD*)	Probability sensitivity analysis values	References
**Transitional probabilities**			
Monthly probability of tracheostomy due to bulbar involvement with Gaucher 2 neonatal-onset disease.	0.0224 ± 0.0020	Inverse beta distribution (*α* = 2.2, *β* = 97.8)	[4]
Monthly probability of tracheostomy due to bulbar involvement with Gaucher 2 infancy-onset disease	0.0224 ± 0.0020	Inverse beta distribution (*α* = 2.2, *β* = 97.8)	[4]
Monthly probability of enteral feeding due to feeding difficulties with Gaucher 2 neonatal-onset disease	0.0443 ± 0.0020	Inverse beta distribution (*α* = 4.4, *β* = 95.6)	[4]
Monthly probability of enteral feeding due to feeding difficulties with Gaucher 2 infancy-onset disease	0.0165 ± 0.0010	Inverse beta distribution (*α* = 4.4, *β* = 95.6)	[5]
Monthly probability of epilepsy with Gaucher 2 neonatal-onset disease.	0.0224 ± 0.0020	Inverse beta distribution (*α* = 2.2, *β* = 97.8)	[4]
Monthly probability of epilepsy with Gaucher 2 infancy-onset disease.	0.0079 ± 0.0001	Inverse beta distribution (*α* = 0.8, *β* = 99.2)	[5]
Monthly probability of interstitial lung disease with Gaucher 2 neonatal-onset disease form	0.0277 ± 0.0034	Inverse beta distribution (*α* = 2.8, *β* = 97.2)	[11]
Monthly probability of interstitial lung disease with Gaucher 2 infancy-onset disease form	0.0277 ± 0.0034	Inverse beta distribution (*α* = 2.8, *β* = 97.2)	[11]
Monthly probability of major bleeding with Gaucher 2 neonatal-onset disease form	0.0324 ± 0.0018	Inverse beta distribution (*α* = 3.2, *β* = 96.8)	[11]
Monthly probability of major bleeding with Gaucher 2 infancy-onset disease form	0.0324 ± 0.0018	Inverse beta distribution (*α* = 3.2, *β* = 96.8)	[11]
Monthly probability of state with multiple complications with Gaucher 2 neonatal-onset disease form	0.0299 ± 0.0020	Inverse beta distribution (*α* = 2.9, *β* = 97.1)	[4,5,11]
Monthly probability of state with multiple complications with Gaucher 2 infancy-onset disease form	0.0214 ± 0.0031	Inverse beta distribution (*α* = 2.1, *β* = 97.9)	[4,5,11]
Monthly probability of death with Gaucher 2 neonatal-onset disease form	0.0274 ± 0.0028	Inverse beta distribution (*α* = 2.7, *β* = 97.3)	[12]
Monthly probability of death with Gaucher 2 infancy-onset disease form	0.0214 ± 0.0019	Inverse beta distribution (*α* = 2.1, *β* = 97.9)	[12]
Death rate after major gastrointestinal bleeding in infants and newborns	0.1 ± 0.05	Inverse beta distribution (*α* = 10, *β* = 90)	[13]
**Effectiveness of active treatment**			
Fraction of probability of neuronal complications when using ambroxol in therapy	0.6670 ± 0.0527	Inverse beta distribution (*α* = 66.7, *β* = 33.3)	[14–17]
**Utilities of health states**			
Utility of state without complications	0.86 ± 0.06	Inverse beta distribution (*α* = 86, *β* = 14)	[18]
Utility of state with tracheostomy	0.68 ± 0.05	Inverse beta distribution (*α* = 68, *β* = 32)	[19]
Utility of state with tube enteral feeding	0.50 ± 0.07	Inverse beta distribution (*α* = 50, *β* = 50)	[20]
Utility decrement of state with epilepsy	0.127 ± 0.023	Inverse beta distribution (*α* = 12.7, *β* = 87.3)	[21]
Utility decrement of state with interstitial lung disease	0.130 ± 0.031	Inverse beta distribution (*α* = 13, *β* = 87)	[22]
Utility decrement of state with major bleeding	0.16 ± 0.04	Inverse beta distribution (*α* = 16, *β* = 84)	[23]
**Costs of health states**			
Annual direct costs of state without complications	88488.00 ± 8672.00 RSD	Inverse gamma distribution (*α* = 16, *β* = 5530.50)	[24]
Initial direct costs of state without complications	3931.00 ± 820,00 RSD	Inverse gamma distribution (*α* = 16, *β* = 245.68)	[25]
Annual direct costs of tracheostomy state:	108236.92 ± 12800.00 RSD	Inverse gamma distribution (*α* = 16, *β* = 6764.81)	[25]
Initial costs of tracheostomy state:	30901.00 ± 3400.00 RSD	Inverse gamma distribution (*α* = 16, *β* = 1931.31)	[25]
Annual direct costs of enteral feeding state	451016.22 ± 62900.00 RSD	Inverse gamma distribution (*α* = 16, *β* = 28188.51)	[26]
Initial direct costs of enteral feeding state	33201.00 ± 4020.00 RSD	Inverse gamma distribution (*α* = 16, *β* = 2075.06)	[27]
Annual direct costs of epilepsy	96338.22 ± 7700.00 RSD	Inverse gamma distribution (*α* = 16, *β* = 1541.41)	[27]
Initial direct costs of epilepsy	6556.69 ± 1100.00 RSD	Inverse gamma distribution (*α* = 16, *β* = 409.79)	[28]
Annual direct costs of interstitial lung disease	108646.77 ± 14060.00 RSD	Inverse gamma distribution (*α* = 16, *β* = 6790.42)	[29]
Initial direct costs of interstitial lung disease	15093.16 ± 1450.00 RSD	Inverse gamma distribution (*α* = 16, *β* = 943.32)	[30]
Monthly direct costs of major bleeding epizode	10574.06 ± 2100.00 RSD	Inverse gamma distribution (*α* = 16, *β* = 660.88)	[31]
**Unit costs of drugs**			
Unit cost of ambroxol (Flavamed): 15 mg/5 ml, oral solution, 100 ml	RSD 600.00	Inverse gamma distribution (*α* = 16, *β* = 37.5)	[32]
Unit cost of Imiglucerase, powder to be reconstituted for infusion; 400j	151702.80 RSD	Inverse gamma distribution (*α* = 16, *β* = 9481.43)	[33]
**Gross domestic product per capita**			
Gross domestic product per capita in Serbia 2021	917441.90 RSD	n.a.	[34]

*SD: standard deviation.

Our major outcomes were quality-adjusted life years (QALYs) gained with ambroxol + imiglucerase and imiglucerase only, and direct costs of treatment. Within direct costs, we have included the costs of drugs, diagnostic and therapeutic healthcare services, materials for healthcare, and costs of diagnosing and treating adverse drug reactions. Given that we conducted the analysis from the perspective of RHIF, as an institution that is the dominant insurance carrier, we took into account only direct costs, while we did not count indirect and intangible costs. We used published cost-effectiveness studies or clinical practice guidelines for the treatment of GD2 for resource utilization. For the determination of unit prices, we used the following sources: the Tariff book of the RHIF (for prices of health services in the Republic of Serbia) [35] and “List of Reimbursable Drugs” of the RHIF [36] or from the “Decision on Maximum Prices of Medicines of the Government of the Republic of Serbia” [33] (for unit prices of drugs). We expressed all costs in the Republic of Serbia Dinars (RSD), which is the official currency of the Republic of Serbia. The estimated resource quantities and unit costs related to year 2022. When converting costs from foreign currency to RSD, the National Bank of Serbia’s middle exchange rate was utilized, which was in effect on the day the primary data were published or collected. To implement the results of our analysis in the future, we introduced a 5% annual discount rate for all costs and QALYs starting from the second year until the end of the horizon [37]. The cost-effectiveness of the therapeutic options was compared by calculating incremental cost-effectiveness ratio (ICER) and comparing it with threshold values, shown as lambda lines at the incremental cost-effectiveness plane. The threshold values were set at 1, 3, and 9 gross domestic products (GDPs) per capita per QALY saved; the lambda 3 line representing the threshold of 9 GDPs/QALY is the upper limit of acceptance by health insurance funds granted to orphan drugs only, taking into account difficulties of developing orphan drugs and limited return of investment due to small size of their market.

**Figure 1:**
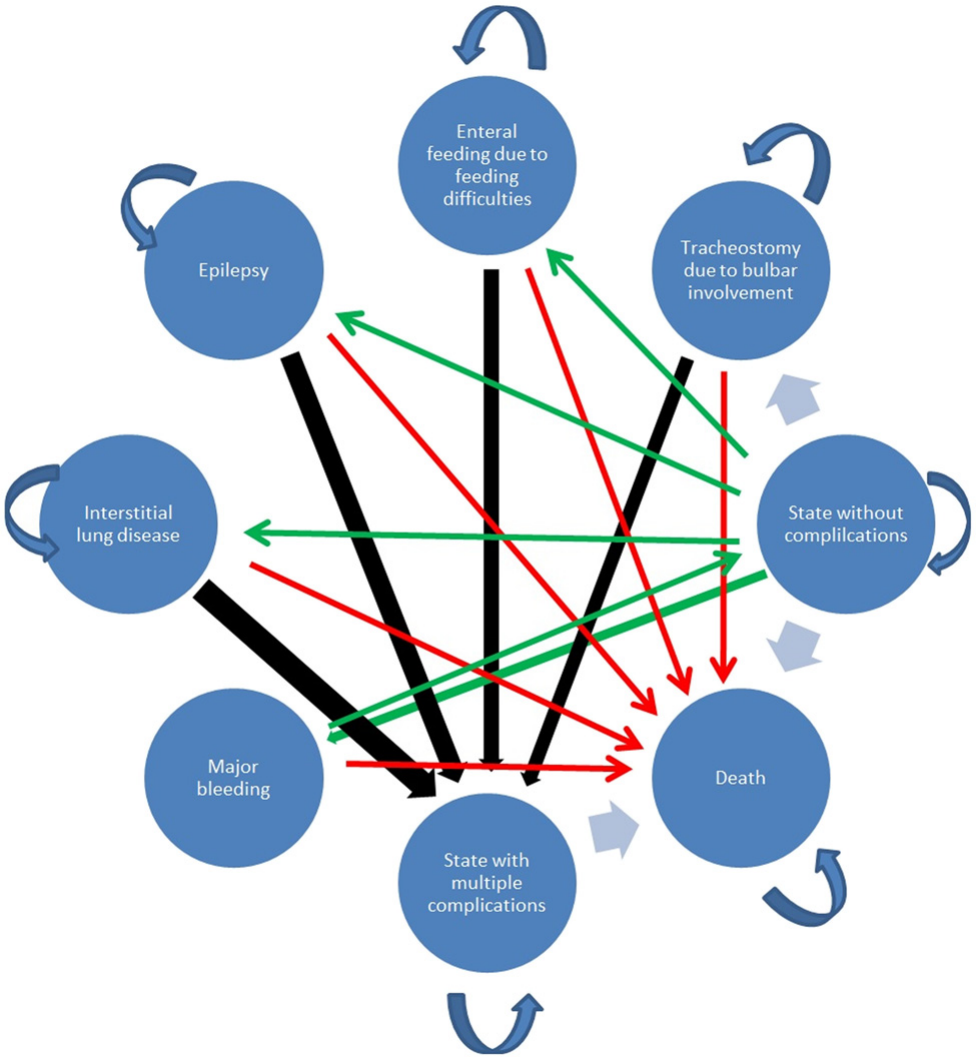
Gaucher disease type 2 model illustrated graphically.

The study was conducted through the generation and simulation of the Markov chain model which describes course of the GD2 through a series of health states [38]. The Markov chain model assumes that a virtual patient may spend any of the monthly cycles in one of the possible clinical states; at the end of each cycle, the patient may stay in the same state, or transit to one of the other available states. After assigning probabilities to each of the possible transitions, the model calculates the probability of being in each state in each cycle. Healthcare utilities and costs of the model states are multiplied by probabilities and then summed up for whole model horizon, giving as output total costs and QALYs for each virtual patient. Monte Carlo simulation uses random number generation to place every virtual patient in a certain state at each model cycle following previously calculated probability distribution [39]. We developed our model in Microsoft Excel, version 2019, while we used Virtual Basic for the development of special macros for the execution of model simulations. A graphic representation of the model is shown in Figure 1.

To test the model’s robustness, we performed both probabilistic and one-way sensitivity analyses. The study population’s heterogeneity was considered by conducting independent analyses on two patient subpopulations, as the rate of progression is directly influenced by whether GD2 was first detected at birth or during infancy.

## 3 Results

### 3.1 Onset of the disease at birth

#### 3.1.1 Base case

The base case Monte Carlo microsimulation for 1,000 virtual patients treated by ambroxol + imiglucerase gave the following results: (1) the average cost per patient was 13151610.58 ± 173703.56 RSD (99% CI) and (2) the average number of QALYs gained 1.46 ± 0.01.

On the other hand, for patients treated by monotherapy of imiglucerase: (1) average cost per patient was 14672000.42 ± 197051.56 RSD (99% CI) and (2) average number of QALYs gained 1.34 ± 0.01.

**Figure 2:**
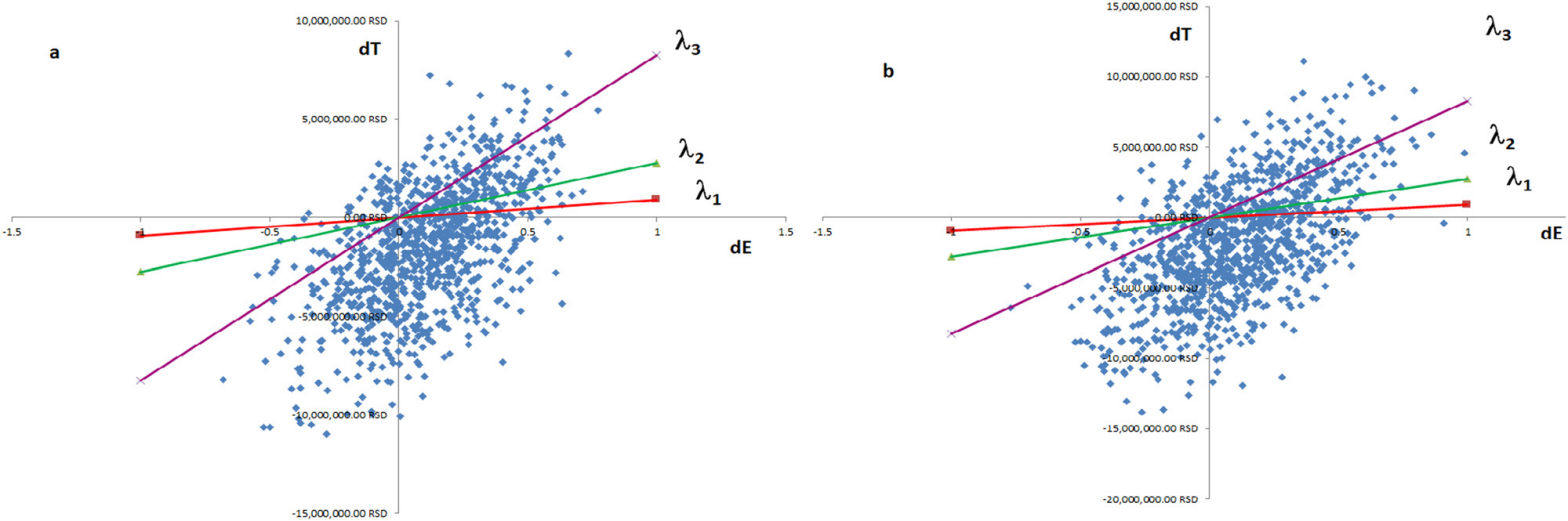
The base case ICERs for virtual patients (a – onset of the disease at birth; b – onset of the disease at infancy). The *x*-axis: difference in QALYs gained (combination of ambroxol and imiglucerase vs imiglucerase only); the *y*-axis: difference in costs (combination of ambroxol and imiglucerase vs imiglucerase only). The line lambda 1 – the RHIF’s willingness to pay one GDP per capita for one more QALY gained with a combination of ambroxol and imiglucerase vs imiglucerase only. The line lambda 2 – the RHIF’s willingness to pay three GDPs per capita for one more QALY gained with a combination of ambroxol and imiglucerase vs imiglucerase only. The line lambda 3 – the RHIF’s willingness to pay nine GDPs per capita for one more QALY gained with a combination of ambroxol and imiglucerase vs imiglucerase only.

In comparison with imiglucerase as monotherapy, the use of a combination of ambroxol and imiglucerase was followed by a positive net monetary benefit (1622511.89 ± 247121.93 RSD [99% CI]), while the value of the ICER per one more QALY gained was 6069649.10 ± 20628071.68 RSD (99% CI). The ICER is displayed separately for each virtual patient in Figure 2a. The combination of ambroxol and imiglucerase was a cost/effective therapeutic option of GD2 evident at birth in comparison to imiglucerase alone.

#### 3.1.2 Acceptability curve

**Figure 3:**
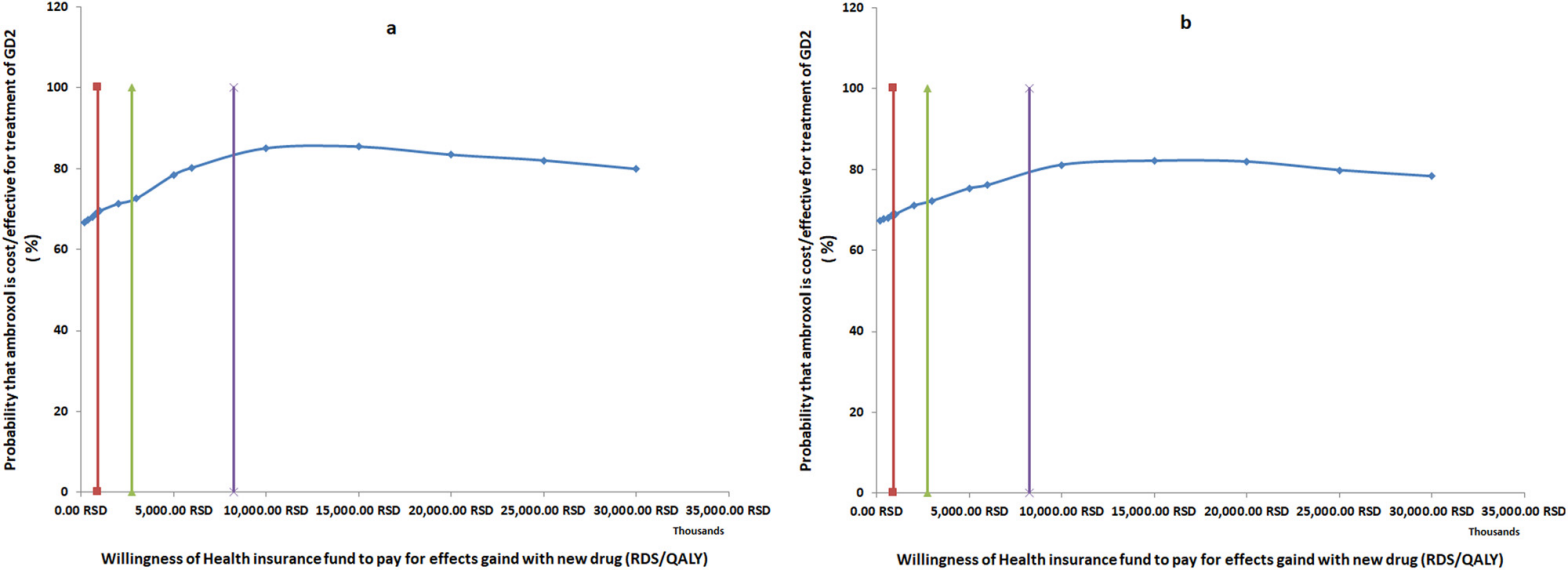
Acceptability curve (a – onset of the disease at birth; b – onset of the disease at infancy).

The acceptability curve reveals the changes in the percentage of patients who fall below the current willingness to pay line in the ICER diagram under the condition that the willingness of RHIF to pay one more QALY gained increases from 200000.00 to 250000000.00 RSD (Figure 3a).

#### 3.1.3 One-way sensitivity analysis

**Figure 4:**
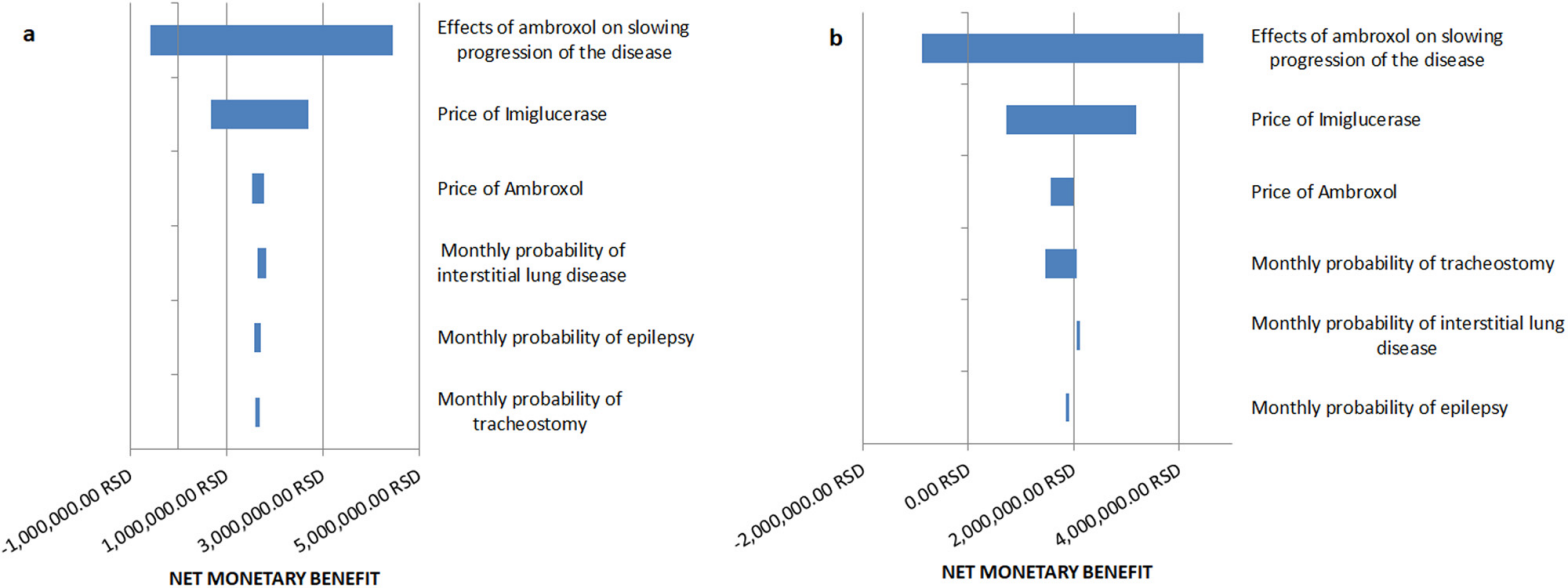
Tornado diagram (a – onset of the disease at birth; b – onset of the disease at infancy).

The input variable values were varied by ±25% each, within the scope of a one-way sensitivity analysis. The net monetary benefit was then computed for each of the varied values. On the Tornado diagrams, we have shown the results for only the six most influential variables to make the graphics as clear as possible (Figure 4a). The results of the one-way sensitivity analysis showed that the effect of ambroxol on slowing the progression of neurological complications of the disease has the greatest impact on its cost-utility and net monetary benefit. Considering other variables, even if they take extreme values in both directions, ambroxol remains cost-effective, since the net monetary benefit stays positive.

#### 3.1.4 Probabilistic sensitivity analysis (PSA)

**Figure 5:**
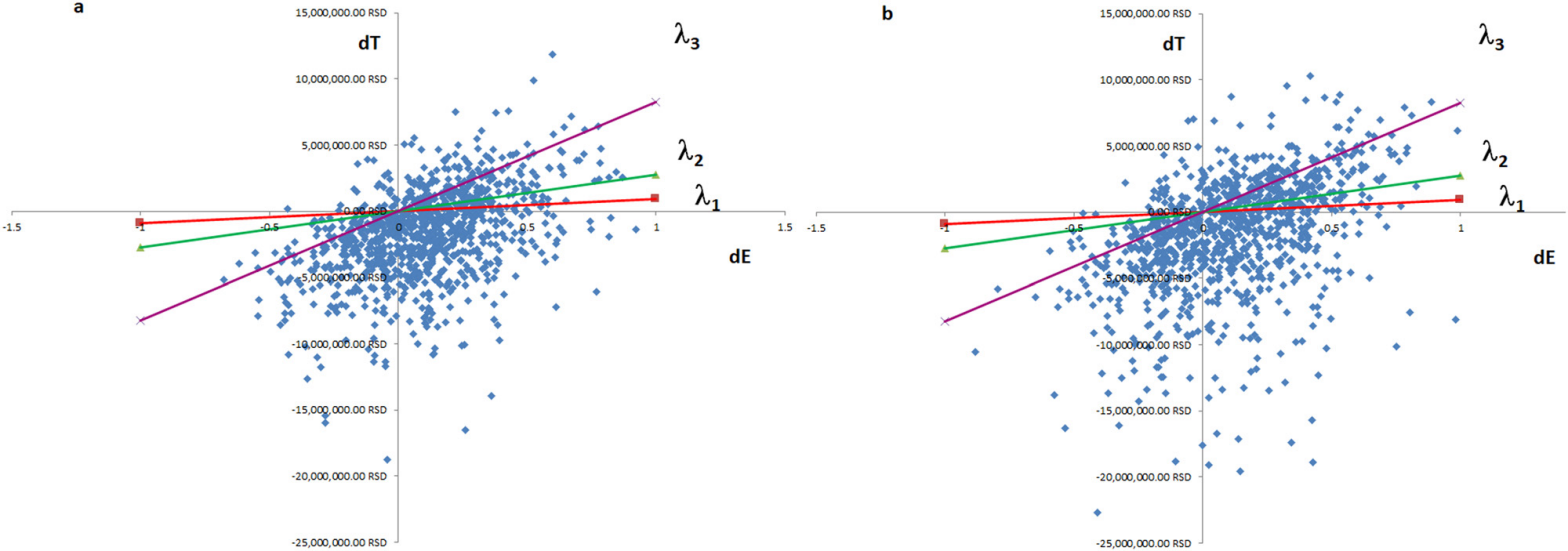
The probability sensitivity analysis ICERs for virtual patients (a – onset of the disease at birth; b – onset of the disease at infancy). The *x*-axis: difference in QALYs gained (combination of ambroxol and imiglucerase vs imiglucerase only); the *y*-axis: difference in costs (combination of ambroxol and imiglucerase vs imiglucerase only). The line lambda 1 – the RHIF’s willingness to pay one GDP per capita for one more QALY gained with a combination of ambroxol and imiglucerase vs imiglucerase only. The line lambda 2 – the RHIF’s willingness to pay three GDPs per capita for one more QALY gained with a combination of ambroxol and imiglucerase vs imiglucerase only. The line lambda 3 – the RHIF’s willingness to pay nine GDPs per capita for one more QALY gained with a combination of ambroxol and imiglucerase vs imiglucerase only.

For the PSA, values of the input variables were replaced with distributions, with the beta distribution being used for rate and utility variables and the gamma distribution for cost variables. After the Monte Carlo microsimulation, similarly dispersed values of output variables were recorded (Figure 5a), and their means with 99% confidence intervals are presented in Table 2. With consistently below-threshold values of ICER and positive values of net monetary benefit, the PSA confirmed that ambroxol in combination with imiglucerase is a cost-effective option for the treatment of GD2 when compared with monotherapy with imiglucerase.

**Table 2:** Values of main output variables before and after the probabilistic sensitivity analysis (mean ± 99% confidence interval)

Output variables	Base case	Probabilistic sensitivity analysis
**Neonatal-onset GD2**		
ICER*	6069649.10 ± 20628071.68 RSD	−651176.53 ± 12563110.24 RSD
Net monetary benefit	1622511.89 ± 247121.93 RSD	1785185.66 ± 271360.12 RSD
**Infancy-onset GD2**		
ICER*	−30202486.52 ± 63597201.79 RSD	−292239212.30 ± 771851457.72 RSD
Net monetary benefit	1888149.51 ± 325318.82 RSD	1764899.06 ± 347175.45 RSD

*ICER: incremental cost-effectiveness ratio.

### 3.2 Onset of the disease in infancy

#### 3.2.1 Base case

The base case Monte Carlo microsimulation for 1,000 virtual patients treated by ambroxol + imiglucerase gave the following results: (1) the average cost per patient was 17071607.90 ± 222453.36 RSD (99% CI) and (2) the average number of QALYs gained 1.58 ± 0.02.

On the other hand, for patients treated by monotherapy of imiglucerase, (1) average cost per patient was 18862126.63 ± 255446.49 RSD (99% CI) and (2) average number of QALYs gained 1.47 ± 0.01.

In comparison with imiglucerase as monotherapy, the use of a combination of ambroxol and imiglucerase was followed by a positive net monetary benefit (1888149.51 ± 325318.82 RSD [99% CI]), while the value of the ICER per one more QALY gained was −30202486.52 ± 63597201.79 RSD (99% CI). The ICER is displayed separately for each virtual patient in Figure 2b. The combination of ambroxol and imiglucerase was a cost/effective therapeutic option for GD2 emerging in infancy in comparison to imiglucerase alone.

#### 3.2.2 Acceptability curve

The acceptability curve reveals the changes in the percentage of patients who fall below the current willingness to pay line in the ICER diagram under the condition that the willingness of RHIF to pay one more QALY gained increases from 200000.00 to 250000000.00 RSD (Figure 3b).

#### 3.2.3 One-way sensitivity analysis

The input variable values were varied by ±25% each, within the scope of a one-way sensitivity analysis. The net monetary benefit was then computed for each of the varied values. On the Tornado diagrams, we have shown the results for only the six most influential variables to make the graphics as clear as possible (Figure 4b). The results of the one-way sensitivity analysis showed that the effect of ambroxol on slowing the progression of neurological complications of the disease has the greatest impact on its cost-utility and net monetary benefit. Considering other variables, even if they take extreme values in both directions, ambroxol remains cost-effective, since the net monetary benefit stays positive.

#### 3.2.4 Probabilistic sensitivity analysis (PSA)

For the PSA, values of the input variables were replaced with distributions, with the beta distribution being used for rate and utility variables and the gamma distribution for cost variables. After the Monte Carlo microsimulation, similarly dispersed values of output variables were recorded (Figure 5b), and their means with 99% confidence intervals are presented in Table 2. With consistently below-threshold values of ICER and positive values of net monetary benefit, the PSA confirmed that ambroxol in combination with imiglucerase is a cost-effective option for the treatment of GD2 when compared with monotherapy with imiglucerase.

## 4 Discussion

Our research revealed that, for the treatment of GD2 in the socioeconomic context of the Republic of Serbia, ambroxol in combination with imiglucerase was more cost-effective than imiglucerase monotherapy. Treatment of GD2 with ambroxol and imiglucerase in combination was clearly more effective and less costly than that with imiglucerase only. Such beneficial result for ambroxol and imiglucerase combination is primarily driven by the low cost of ambroxol and its considerable clinical effectiveness in slowing the progression of neural complications of GD2.

Ambroxol is not officially approved for the treatment of GD2 [8,40]. It was identified in an experimental study from 2009 as a credible contender for a pharmacological chaperone for mutant glucocerebrosidase [41]. In 2016, a pilot study investigating the efficacy and safety of ambroxol combined with imiglucerase in patients with neurological manifestations of Gaucher disease (GD2 and GD3) was published [6]. The results of this study showed that the use of high doses of ambroxol in combination with enzyme replacement therapy was accompanied by satisfactory safety and clinical efficacy in patients suffering from neurological types of Gaucher’s disease, since there was a significant improvement in myoclonus, a reduction in the frequency of epileptic seizures, and an improvement in other neurological manifestations [6].

Nonetheless, despite the encouraging preliminary results, it seems that pharmaceutical companies are not very interested in looking into whether ambroxol may be used to treat patients with neurological forms of GD [42]. It is believed that pharmaceutical companies are not particularly interested in continuing clinical trials examining the efficacy of amborxol in patients with GD2 and GD3 due to amborxol’s low costs and limited potential for revenue [32]. This kind of approach by pharmaceutical companies has far-reaching consequences. First of all, this puts in jeopardy the fundamental humanitarian and egalitarian ideals that state that every patient has a right to the best medical care. [43]. In addition, pharmaceutical companies that develop orphan drugs enjoy various tax and other incentives, which are missed in this case [44].

Meanwhile, ambroxol is reported to be used off-label in combination with enzyme replacement therapy for the treatment of children with GD2 and GD3 [14]. Particularly, promising results came from a girl with GD2 who got ambroxol monotherapy at a daily dose of 25 mg/kg beginning at the end of her third month of life [8]. The achieved effect of this therapy was impressive, since at the end of her first year of life; this girl achieved the neurocognitive and motor development that is expected for her age [8]. Imiglucerase was introduced into therapy only at the 15th month of life, even 11 months after the administration of ambroxol [8]. The combined use of ambroxol and imiglucerase had a long-lasting effect because the girl’s good neurocognitive growth and satisfactory quality of life persisted even at age 3 [8]. The results of our study, which showed the financial justification of using ambroxol in combination with imiglucerase in GD patients, should be a major impetus for parents of affected children as well as associations and foundations for rare diseases to exert the necessary pressure and raise the issue of the continuation of clinical trials.

Our study was limited by the fact that ambroxol is available over the counter and is not covered by the Republic Health Insurance Fund; as a result, we were forced to use the rates set by local vendors because Serbia did not yet have an official maximum price for this medication.

## 5 Conclusion

With the current price, ambroxol in combination with enzyme replacement therapy is a cost-effective option in comparison to the therapy of GD2 with only enzyme replacement. Considering the encouraging results of a growing number of individual patient cases that indicate satisfactory efficacy and safety of ambroxol as an additional therapy, the health insurance fund should reimburse ambroxol for this indication to all patients with this disease once the drug is officially approved for this indication. The findings of our study might encourage regulatory agencies and organizations for rare diseases to persuade pharmaceutical companies to continue clinical trials of ambroxol in patients with GD2.

## References

[1] Stirnemann J, Belmatoug N, Camou F, Serratrice C, Froissart R, Caillaud C, et al. A review of Gaucher disease pathophysiology, clinical presentation and treatments. Int J Mol Sci. 2017;18(2):441. 10.3390/ijms18020441 PMC5343975 28218669

[2] Roh J, Subramanian S, Weinreb NJ, Kartha RV. Gaucher disease – more than just a rare lipid storage disease. J Mol Med. 2022;100(4):499–518. 10.1007/s00109-021-02174-z 35066608

[3] Weiss K, Gonzalez A, Lopez G, Pedoeim L, Groden C, Sidransky E. The clinical management of Type 2 Gaucher disease. Mol Genet Metab. 2015;114(2):110–22. 10.1016/j.ymgme.2014.11.008 PMC4312716 25435509

[4] Roshan Lal T, Seehra GK, Steward AM, Poffenberger CN, Ryan E, Tayebi N, et al. The natural history of type 2 Gaucher disease in the 21st century: A retrospective study. Neurology. 2020;95(15):e2119–30. 10.1212/WNL.0000000000010605 PMC7713752 32764102

[5] Tylki-Szymańska A, Vellodi A, El-Beshlawy A, Cole JA, Kolodny E. Neuronopathic Gaucher disease: Demographic and clinical features of 131 patients enrolled in the International Collaborative Gaucher Group Neurological Outcomes Subregistry. J Inherit Metab Dis. 2010;33(4):339–46. 10.1007/s10545-009-9009-6 20084461

[6] Narita A, Shirai K, Itamura S, Matsuda A, Ishihara A, Matsushita K, et al. Ambroxol chaperone therapy for neuronopathic Gaucher disease: A pilot study. Ann Clin Transl Neurol. 2016;3(3):200–15. 10.1002/acn3.292 PMC4774255 27042680

[7] World Bank. Upper middle income | Data. [cited 2023 Mar 23]. https://data.worldbank.org/income-level/upper-middle-income.

[8] Aries C, Lohmöller B, Tiede S, Täuber K, Hartmann G, Rudolph C, et al. Promising effect of high dose ambroxol treatment on neurocognition and motor development in a patient with neuropathic Gaucher disease 2. Front Neurol. 2022;13:907317. 10.3389/fneur.2022.907317 PMC9207411 35734474

[9] Republic Fund of Health Insurance. [cited 2023 Mar 23]. https://www.rfzo.rs/.

[10] Gupta N, Oppenheim IM, Kauvar EF, Tayebi N, Sidransky E. Type 2 Gaucher disease: phenotypic variation and genotypic heterogeneity. Blood Cell Mol Dis. 2011;46(1):75–84. 10.1016/j.bcmd.2010.08.012 PMC3018671 20880730

[11] Mignot C, Doummar D, Maire I, De Villemeur TB. French type 2 Gaucher Disease Study Group. Type 2 Gaucher disease: 15 New cases and review of the literature. Brain Dev. 2006;28(1):39–48. 10.1016/j.braindev.2005.04.005 16485335

[12] Nalysnyk L, Rotella P, Simeone JC, Hamed A, Weinreb N. Gaucher disease epidemiology and natural history: A comprehensive review of the literature. Hematol Amst Neth. 2017;22(2):65–73. 10.1080/10245332.2016.1240391 27762169

[13] Romano C, Oliva S, Martellossi S, Miele E, Arrigo S, Graziani MG, et al. Pediatric gastrointestinal bleeding: Perspectives from the Italian Society of Pediatric Gastroenterology. World J Gastroenterol. 2017;23(8):1328–37. 10.3748/wjg.v23.i8.1328 PMC5330817 28293079

[14] Istaiti M, Revel-Vilk S, Becker-Cohen M, Dinur T, Ramaswami U, Castillo-Garcia D, et al. Upgrading the evidence for the use of ambroxol in Gaucher disease and GBA related Parkinson: Investigator initiated registry based on real life data. Am J Hematol. 2021;96(5):545–51. 10.1002/ajh.26131 33606887

[15] Mohamed FE, Al-Jasmi F. Exploring the efficacy and safety of Ambroxol in Gaucher disease: an overview of clinical studies. Front Pharmacol. 2024;15:1335058. 10.3389/fphar.2024.1335058 PMC10896849 38414738

[16] Zhan X, Zhang H, Maegawa GHB, Wang Y, Gao X, Wang D, et al. Use of ambroxol as therapy for Gaucher Disease. JAMA Netw Open. 2023;6(6):e2319364. 10.1001/jamanetworkopen.2023.19364 PMC10285580 37342037

[17] Weinreb NJ, Goker-Alpan O. Ambroxol as therapy for Gaucher disease-ambitious but ambivalent. JAMA Netw Open. 2023;6(6):e2319336. 10.1001/jamanetworkopen.2023.19336 37342045

[18] Connock M, Burls A, Frew E, Fry-Smith A, Juarez-Garcia A, McCabe C, et al. The clinical effectiveness and cost-effectiveness of enzyme replacement therapy for Gaucher’s disease: A systematic review. Health Technol Assess Winch Engl. 2006;10(24):iii–iv, ix–136. 10.3310/hta10240 16796930

[19] Naunheim MR, Song PC, Franco RA, Alkire BC, Shrime MG. Surgical management of bilateral vocal fold paralysis: A cost-effectiveness comparison of two treatments. Laryngoscope. 2017;127(3):691–7. 10.1002/lary.26253 27578299

[20] Hausmann J, Kubesch A, Goettlich CM, Rey J, Wächtershäuser A, Bojunga J, et al. Quality of life of patients with head and neck cancer after prophylactic percutaneous-gastrostomy. Eur J Clin Nutr. 2020;74(4):565–72. 10.1038/s41430-019-0499-5 31570758

[21] Lee DC, Gladwell D, Hatswell AJ, Porter J, Brereton N, Tate E, et al. A comparison of the cost-effectiveness of treatment of prolonged acute convulsive epileptic seizures in children across Europe. Health Econ Rev. 2014;4:6. 10.1186/s13561-014-0006-6 PMC4052771 24949280

[22] Lauby C, Boelle PY, Abou Taam R, Bessaci K, Brouard J, Dalphin ML, et al. Health-related quality of life in infants and children with interstitial lung disease. Pediatr Pulmonol. 2019;54(6):828–36. 10.1002/ppul.24308 30868755

[23] Henry N, Jovanović J, Schlueter M, Kritikou P, Wilson K, Myrén KJ. Cost-utility analysis of life-long prophylaxis with recombinant factor VIIIFc vs recombinant factor VIII for the management of severe hemophilia A in Sweden. J Med Econ. 2018;21(4):318–25. 10.1080/13696998.2017.1405816 29139314

[24] Qi X, Xu J, Shan L, Li Y, Cui Y, Liu H, et al. Economic burden and health related quality of life of ultra-rare Gaucher disease in China. Orphanet J Rare Dis. 2021;16(1):358. 10.1186/s13023-021-01963-6 PMC8356434 34380529

[25] Mitchell RB, Hussey HM, Setzen G, Jacobs IN, Nussenbaum B, Dawson C, et al. Clinical consensus statement: tracheostomy care. Otolaryngol–Head Neck Surg J Am Acad Otolaryngol–Head Neck Surg. 2013;148(1):6–20. 10.1177/0194599812460376 22990518

[26] Mehta NM, Skillman HE, Irving SY, Coss-Bu JA, Vermilyea S, Farrington EA, et al. Guidelines for the provision and assessment of nutrition support therapy in the pediatric critically ill patient: Society of critical care medicine and American society for parenteral and enteral nutrition. JPEN J Parenter Enter Nutr. 2017;41(5):706–42. 10.1177/0148607117711387 28686844

[27] Blumenstein I, Shastri YM, Stein J. Gastroenteric tube feeding: techniques, problems and solutions. World J Gastroenterol. 2014;20(26):8505–24. 10.3748/wjg.v20.i26.8505 PMC4093701 25024606

[28] Cramer JA, Wang ZJ, Chang E, Powers A, Copher R, Cherepanov D, et al. Healthcare utilization and costs in children with stable and uncontrolled epilepsy. Epilepsy Behav EB. 2014;32:135–41. 10.1016/j.yebeh.2014.01.016 24561658

[29] Szentes B, Witt S, Bush A, Cunningham S, Emiralioğlu N, Goldbeck L, et al. Healthcare utilisation in childhood interstitial lung diseases: Analysis of chILD-EU registry data. Eur Respir J. 2016;48(suppl 60):PA4248. [cited 2022 Dec 15]. https://erj.ersjournals.com/content/48/suppl_60/PA4248.

[30] Bush A, Cunningham S, de Blic J, Barbato A, Clement A, Epaud R, et al. European protocols for the diagnosis and initial treatment of interstitial lung disease in children. Thorax. 2015;70(11):1078–84. 10.1136/thoraxjnl-2015-207349 26135832

[31] McEvoy MT, Shander A. Anemia, bleeding, and blood transfusion in the intensive care unit: causes, risks, costs, and new strategies. Am J Crit Care Publ Am Assoc Crit-Care Nurses. 2013;22(6 Suppl):eS1–13. quiz eS14. 10.4037/ajcc2013729 24186829

[32] Gospodar zdravlja. Flavamed sirup za iskašljavanje – upotreba, iskustva i cena. [cited 2023 Mar 23]. 2017. https://gospodarzdravlja.com/flavamed-sirup-za-iskasljavanje-upotreba-iskustva-i-cena/.

[33] The Government of the Republic of Serbia. Decision on the highest prices of prescription medicines for human use. Off Gaz RS. 48/2021; 2021.

[34] Statistical Office of the Republic of Serbia; 2022. [cited 2023 Mar 23]. https://data.stat.gov.rs/Home/Result/09020101?languageCode=sr-Latn.

[35] Republic Health Insurance Fund of Serbia. Rulebook on the prices of health services at the secondary and tertiary levels of health care. 2021. Off Gaz RS. 55/2019, 53/2021.

[36] Republic Health Insurance Fund of Serbia. Rulebook on the list of medicines prescribed and issued at the expense of compulsory health insurance funds. 2021. Off Gaz RS. 43/19, 55/19, 56/19-correction, 73/19, 87/19, 18/20, 43/20, 108/20, 49/21, 51/21-correction and 60/21.

[37] National Bank of Serbia. Interest rates. [cited 2023 Mar 23]. 2023. https://www.nbs.rs/sr/ciljevi-i-funkcije/monetarna-politika/kamatne-stope/.

[38] Green N, Lamrock F, Naylor N, Williams J, Briggs A. Health economic evaluation using markov models in R for microsoft excel users: A tutorial. PharmacoEconomics. 2022;41(1):5–19. 10.1007/s40273-022-01199-7 36336774

[39] Rui M, Wang Y, Fei Z, Zhang X, Shang Y, Li H. Will the Markov model and partitioned survival model lead to different results? A review of recent economic evidence of cancer treatments. Expert Rev Pharmacoecon Outcomes Res. 2021;21(3):373–80. 10.1080/14737167.2021.1893167 33691544

[40] Bouscary A, Quessada C, René F, Spedding M, Henriques A, Ngo S, et al. Drug repositioning in neurodegeneration: An overview of the use of ambroxol in neurodegenerative diseases. Eur J Pharmacol. 2020;884:173446. 10.1016/j.ejphar.2020.173446 32739173

[41] Maegawa GH, Tropak MB, Buttner JD, Rigat BA, Fuller M, Pandit D, et al. Identification and characterization of ambroxol as an enzyme enhancement agent for Gaucher disease. J Biol Chem. 2009;284:23502–16. 10.1074/jbc.M109.012393 PMC2749124 19578116

[42] ClinicalTrials.gov. World Data on Ambroxol for Patients With GD and GBA Related PD (NCT04388969). [cited 2023 Mar 23]. 2020. https://clinicaltrials.gov/ct2/show/NCT04388969?cond=ambroxol&draw=2&rank=3.

[43] Iskrov G, Stefanov R. Criteria for drug reimbursement decision-making: An emerging public health challenge in Bulgaria. Balk Med J. 2016;33(1):27–35. 10.5152/balkanmedj.2015.15185 PMC4767306 26966615

[44] Murphy SM, Puwanant A, Griggs RC. Consortium for clinical investigations of neurological channelopathies (CINCH) and inherited neuropathies consortium (INC) consortia of the rare disease clinical research network. Unintended effects of orphan product designation for rare neurological diseases. Ann Neurol. 2012;72(4):481–90. 10.1002/ana.23672 PMC3490440 23109143

